# Effects of Disease-Related Knowledge on Illness Perception and Psychological Status of Patients With COVID-19 in Hunan, China

**DOI:** 10.1017/dmp.2021.33

**Published:** 2021-02-16

**Authors:** Man Ye, Shi-hao Chen, Xu-ting Li, Jin Huang, Ran-ran Mei, Tie-ying Qiu, Ya-min Li, Hui-lin Zhang, Qiong-ni Chen, Chao-ying Xie, Yan-hua Cheng, Jian-wei Zhou

**Affiliations:** 1 Clinical Nursing Teaching and Research Section, The Second Xiangya Hospital, Central South University, Changsha, Hunan, China; 2 Department of Thoracic Surgery, The Second Xiangya Hospital, Central South University, Changsha, Hunan, China; 3 Xiangya Nursing School of Central South University, Changsha, Hunan, China; 4 Changsha Public Health Treatment Center, Hunan, China; 5 The First People’s Hospital of Yueyang, Hunan, China; 6 The Second People’s Hospital of Yueyang, Hunan, China

**Keywords:** COVID-19, knowledge, illness perception, psychological state

## Abstract

**Objective:**

The aim of this study was to assess the current status of disease-related knowledge and to analyze the relationship among the general condition, illness perception, and psychological status of patients with coronavirus disease 2019 (COVID-19).

**Methods:**

A hospital-based cross-sectional study was conducted on 118 patients using convenience sampling. The general questionnaire, disease-related knowledge questionnaire of COVID-19, Illness Perception Questionnaire (IPQ), and Profile of Mood States (POMS) were used to measure the current status of participants.

**Results:**

The overall average score of the disease-related knowledge of patients with COVID-19 was (79.19 ± 14.25), the self-care situation was positively correlated with knowledge of prevention and control (*r* = 0.265; *P* = 0.004) and total score of disease-related knowledge (*r* = 0.206; *P* = 0.025); the degree of anxiety was negatively correlated with the knowledge of diagnosis and treatment (*r* = −0.182; *P* = 0.049). The score of disease-related knowledge was negatively correlated with negative cognition (volatility, consequences, emotional statements) and negative emotions (tension, fatigue, depression) (*P* < 0.05); positively correlated with positive cognition (disease coherence) and positive emotion (self-esteem) (*P* < 0.05).

**Conclusions:**

It was recommended that we should pay more attention to the elderly and low-income groups, and increase the knowledge about diagnosis and treatment of COVID-19 and self-care in the future health education for patients.

The 2019 novel coronavirus (2019-nCoV) was discovered in Wuhan in December 2019, and the disease spread rapidly in other provinces in China,^1^ which was subsequently named novel coronavirus disease 2019 (COVID-19). According to data released by the National Health Commission of China, as of March 10, 2020, the number of confirmed cases in mainland China has increased to 80,778, with a total of 3158 deaths.^2^ In addition, human-to-human transmission has also occurred in many countries and regions outside mainland China.^3^ As of March 12, there were 114 countries in the world with hundreds of thousands of people infected with the disease and WHO defines it was a global pandemic.^4^ The 2019-nCoV was also known as severe acute respiratory syndrome coronavirus (SARS-CoV-2), which was mainly transmitted by means of respiratory droplets and contact, and the population was generally more susceptible than the SARS virus that broke out in 2003.^5^ The clinical symptoms of COVID-19 include headache, fever, cough, muscle pain, dyspnea, hemoptysis fatigue, and diarrhea.^6^ Due to the lack of targeted and effective treatments, the best way to deal with the novel coronavirus pneumonia pandemic is to control the source of infection, cut off the transmission route, and protect vulnerable populations. Specific strategies include strengthening hand hygiene and wearing masks, avoiding large gatherings, tracking the health of company employees, disinfecting the workplace, stopping the use of central air conditioning and enhancing indoor ventilation, encouraging people to exercise regularly, and ensuring adequate rest.^7^ People’s knowledge of disease-related prevention and control COVID-19 would affect the effectiveness of self-protection measures to a certain extent.

Since the outbreak of the COVID-19 epidemic, the Chinese government has quickly adopted response measures and has taken travel-restrictive actions in many provinces and cities to curb the spread of the virus.^8^ Many people isolated themselves at home to prevent infection, and some people gradually developed panic symptoms, depression, anxiety, stress, posttraumatic stress symptoms, and insomnia.^9-11^ Because the COVID-19 epidemic has no specific vaccine and can only be treated symptomatically, this has become a major source of stress for diagnosed patients with COVID-19. Previous studies have shown that patients with large infectious diseases, such as SARS, can produce varying degrees of anxiety, depression, stress, and posttraumatic stress disorder, which severely affect patients’ rehabilitation and quality of life.^12,13^

Disease-related knowledge is very important for patients, it can improve treatment compliance and mental health.^14,15^ Previous studies have shown that lack of knowledge or health information related to COVID-19 can increased patients’ anxiety and depression, and severely reduce their mental health.^9,16^ Improving disease-related knowledge would reduce patient anxiety, improve treatment compliance and satisfaction, and reduce treatment costs.^9,14^ Obtaining appropriate disease-related knowledge could enable patients to effectively cope with themselves before they visit a doctor.^17^ Illness perception is the patient’s view of the current disease. It affected the patient’s emotions, coping strategies, treatment compliance, and disease outcomes.^18,19^ Previous studies have shown that illness perception was constrained by multiple factors, such as the cause of the disease, clinical symptoms, and prognosis.^19^

The ongoing COVID-19 epidemic is spreading globally, and maintaining the healthy mental health of patients is an urgent need for society. However, few studies have been conducted on disease-related knowledge and its effects on illness perception and mental state in patients with COVID-19. The purpose of this study was to assess the current status of disease-related knowledge of patients with COVID-19 and to analyze its relationship among the general condition, illness perception, and psychological status of patients. This study may provide a good reference for government agencies and health-care professionals in China and other countries or regions to effectively protect the mental health of diagnosed patients and other people in the face of the expanding COVID-19 epidemic.

## Methods

### Setting and Participants

This study was a cross-sectional design. A total of 118 inpatients with COVID-19 at the designated hospitals in Hunan Province in February 2020 were selected for this study. Inclusion criteria: (1) patients diagnosed according to the COVID-19 diagnosis and treatment plan (Trial Version-5); (2) over 18 y of age; (3) normal reading and writing ability, understand the questionnaire content; (4) can use WeChat-related functions correctly; (5) informed consent, and voluntarily participated in this study. Exclusion criteria: (1) patients with severe mental disorders; (2) patients with organic brain lesions and malignant tumors.

### Procedure

Due to the highly contagious nature of COVID-19, to avoid spreading the virus during the filling and recycling of paper questionnaires, this study used on-site recruitment and guidance, and filled out the questionnaires online for data collection. All patients used WeChat to scan the quick response (QR) code on the website and filled out the survey form. Each participant completed this cross-sectional survey with the help of 2 trained research nurses. The questionnaire was completed in accordance with uniform guidelines. A total of 118 questionnaires were distributed, and all of them were recovered in this study. The effective recovery rate was 100%.

### Ethical Considerations

This study was approved by the Medical Ethics Committee of the Second Xiangya Hospital of Central South University (Approval Number: 2020015), which was consistent with the principles embodied in the Declaration of Helsinki. Researchers explained the purpose and significance of the study to patients before the survey. All participants provided informed consent.

### Measurements

#### General Information Questionnaire

It was designed by the researcher, including sociodemographic data (age, gender, education, occupation, marital status, etc.) and the general condition of the patient (self-care ability, appetite, endurance of activity, sleep, mood condition, negative effects of disease, etc.).

#### Disease-Related Knowledge Questionnaire of COVID-19

The questionnaire (see supplementary material) was designed by researchers based on the COVID-19 diagnosis and treatment plan (Trial Version-5). There were 25 items in the questionnaire, of which 13 items were diagnosis and treatment knowledge (etiological characteristics, epidemiological characteristics, clinical manifestations and classification, diagnosis, treatment, nursing, prognosis, and discharge criteria of the disease, etc.) and 12 items of prevention and control knowledge (How to do home isolation, personal protection, daily cleaning and sanitation, environmental and article cleaning management, etc.). Each item was designed as a single-choice question. The correct answer was 4 points, and the incorrect answer was not counted. The highest score on the questionnaire was 100 points. The higher the score, the better the knowledge level. In this study, the overall questionnaire Cronbach’s α was 0.767, the Cronbach’s α of diagnosis and treatment knowledge dimension was 0.638, and the Cronbach’s α of prevention and control knowledge dimension was 0.632. The overall reliability was good.

#### Illness Perception Questionnaire

The Illness Perception Question (IPQ) was developed by Weinman et al.^20^ and revised by Moss-Morris et al.^21^ The Chinese version designed by Xiong et al.^22^ was used to investigate the illness perception in this study. The scale was divided into 3 parts: symptoms identity, illness representation, and causes. In this study, only the illness representation part was selected to evaluate COVID-19 patients. It included 7 dimensions, including course of disease, volatility, consequences, personal control, treatment control, disease coherence, and emotional statement, with a total of 38 items. All items were scored on a 5-point Likert scale (1 = strongly disagree; 5 = strongly agree). The Cronbach’s α of each factor of the illness representation questionnaire fluctuated from 0.80 to 0.91, and the reliability and validity were good.^22^

#### Profile of Mood States

The POMS was developed by McNair in 1971,^23^ and the Chinese version was translated and revised by Zhu.^24^ There were 40 items on the scale, which were divided into 7 dimensions of tension, anger, fatigue, depression, vigor, confusion, and self-esteem. Each item is scored on a scale of 0 to 4 (0 = never; 4 = almost always), the higher the energy and self-esteem dimensions, the better the emotional state, and the higher scores in the other 5 dimensions, the worse the emotional state. Total Mood Disturbance (TMD) = total negative emotion score (tension, anger, fatigue, depression, confusion)-total positive emotion score (vigor, self-esteem) +100. A higher TMD score indicated a more negative emotional state. The scale had good reliability and validity, and can accurately reflect the psychological state of the subjects.^25^

### Statistical Analysis

SPSS 20.0 software was adopted to analyze the data. Count data were expressed by frequency and percentage, measurement data were described by mean and standard deviation (¯x ± SD), and analysis of variance (ANOVA) was used to compare the differences in disease-related knowledge among patients in different groups; Spearman correlation analysis was used to explore the correlation between disease-related knowledge and illness perception and psychological status. The level of statistical significance was *P* < 0.05.

## Results

### Current Status of Disease-Related Knowledge in Patients With COVID-19

#### Overall Status of COVID-19 Disease-Related Knowledge Scores

The disease diagnosis and treatment knowledge of patients with COVID-19 was (39.29 ± 8.82), the score of disease prevention and control knowledge was (39.90 ± 7.14), and the total score of disease-related knowledge was (79.19 ± 14.25), which was at a moderately high level. However, there were still some patients with low level of knowledge, manifested as a minimum of 20 points for the total score of disease-related knowledge, 12 points for prevention and control knowledge, and 8 points for disease diagnosis and treatment. The results were shown in Table 1.

**Table 1.** Score of disease-related knowledge of COVID-19 (*n* = 118)

Variable	Minimum	Maximum	Mean	SD
Total score	20	96	79.19	14.25
Disease prevention and control	12	48	39.9	7.14
Disease diagnosis and treatment	8	52	39.29	8.82

#### Disease-Related Knowledge of COVID-19 in Different Demographic Characteristics

ANOVA analysis showed that in terms of disease diagnosis and treatment knowledge, there were significant differences in the COVID-19 patients of different age (*F* = 8.681; *P* = 0.000), the self-conceived possibility of being infected before see the doctor (*F* = 4.627; *P* = 0.002), education level (*F* = 7.984; *P* = 0.000), and monthly income (*F* = 2.747; *P* = 0.022). In terms of disease prevention and control knowledge, there were significant differences in the COVID-19 patients of different age (*F* = 4.659; *P* = 0.004), the self-conceived possibility of being-infected before see the doctor (*F* = 3.504; *P* = 0.010), and the attention to the publicity of COVID-19 (*F* = 3.373; *P* = 0.038). In terms of the total score of disease-related knowledge, there were significant differences in the COVID-19 patients of different age (*F* = 8.548; *P* = 0.000), the self-conceived possibility of being-infected before see the doctor (*F* = 4.887; *P* = 0.001), education level (*F* = 4.964; *P* = 0.003), monthly income (*F* = 2.940; *P* = 0.016), and the attention to the publicity of COVID-19 (*F* = 3.107; *P* = 0.049).

The SNK analysis showed that patients who were 31 to 45 y old and who had a high self-conceived possibility of being-infected before see the doctor have higher scores in disease diagnosis and treatment knowledge, disease prevention and control knowledge, and total disease-related knowledge than those in other groups (*P* < 0.01). Patients with a bachelor’s degree or above and a better monthly income showed higher scores in disease diagnosis and treatment knowledge and total disease-related knowledge (*P* < 0.01). Patients with higher attention to the publicity of COVID-19 have showed better scores in disease prevention and control knowledge and total disease-related knowledge (*P* < 0.05) (Table 2).

**Table 2.** Characteristics and disease-related knowledge in COVID-19 patients (*n* = 118)

Variable	Group	*N*	Disease diagnosis and treatment	Disease prevention and control	Total disease-related knowledge
Gender	Female	57	39.79 ± 8.23	39.37 ± 6.88	79.16 ± 13.02
	Male	61	38.82 ± 9.39	40.39 ± 7.40	79.21 ± 15.42
	*F*		0.354	0.605	0.000
	*p*		0.553	0.438	0.983
Age (y)	≦30	20	39.20 ± 7.52	40.80 ± 5.29	80.00 ± 10.70
	31∼45	60	42.53 ± 6.46	41.73 ± 5.67	84.27 ± 9.63
	46∼60	24	35.33 ± 9.49	36.67 ± 8.31	72.00 ± 17.01
	>60	14	32.29 ± 11.79	36.29 ± 9.98	68.57 ± 19.77
	*F*		8.681	4.659	8.548
	*p*		<0.001	0.004	<0.001
Education level	Middle school and below	20	34.20 ± 12.00	37.60 ± 8.65	71.80 ± 19.79
	High school	34	37.18 ± 6.27	39.41 ± 7.51	76.59 ± 12.07
	College degree	25	38.72 ± 9.22	40.48 ± 8.19	79.20 ± 15.92
	Bachelor degree or above	39	44.10 ± 6.19	41.13 ± 4.85	85.23 ± 8.42
	*F*		7.984	1.190	4.964
	*p*		<0.001	0.317	0.003
Profession	Worker	21	36.38 ± 6.31	40.57 ± 5.70	76.95 ± 10.35
	Farmer	1	48.00 ± 0.00	44.00 ± 0.00	92.00 ± 0.00
	Civil servant	7	37.71 ± 11.51	42.29 ± 5.09	80.00 ± 14.05
	Teacher	6	46.00 ± 4.20	44.67 ± 3.93	90.67 ± 6.02
	Medical staff	3	34.67 ± 19.73	34.67 ± 16.65	69.33 ± 36.30
	Freelancer	17	39.53 ± 9.99	39.06 ± 8.89	78.59 ± 18.11
	Jobless	14	36.86 ± 9.82	40.57 ± 7.82	77.43 ± 16.87
	Retirement	9	36.89 ± 8.89	37.78 ± 6.04	74.67 ± 13.56
	Others	40	41.50 ± 7.56	39.30 ± 6.83	80.80 ± 11.89
	*F*		1.561	0.869	1.045
	*p*		0.145	0.545	0.407
Marital status	Unmarried or others	15	39.47 ± 5.83	40.00 ± 6.05	79.47 ± 9.05
	Married	103	39.26 ± 9.20	39.88 ± 7.31	79.15 ± 14.89
	*F*		0.007	0.003	0.007
	*p*		0.934	0.934	0.935
Family income (RMB Yuan)	<500	3	34.67 ± 14.05	36.00 ± 6.93	70.67 ± 20.13
	500∼	2	22.00 ± 8.49	28.00 ± 5.66	50.00 ± 14.14
	1000∼	12	39.33 ± 7.60	39.33 ± 6.57	78.67 ± 11.86
	2000∼	18	36.22 ± 10.93	38.00 ± 11.00	74.22 ± 20.82
	3000∼	36	39.78 ± 7.65	41.56 ± 5.68	81.33 ± 10.86
	≧5000	47	41.11 ± 7.99	40.26 ± 6.04	81.36 ± 12.29
	*F*		2.747	2.059	2.940
	*p*		0.022	0.076	0.016
Address	Urban area	105	39.58 ± 8.87	38.89 ± 7.24	79.47 ± 14.42
	Township	8	37.50 ± 5.63	40.50 ± 6.57	78.00 ± 9.80
	Rural area	5	36.00 ± 12.33	39.20 ± 7.16	75.20 ± 18.42
	*F*		0.565	0.052	0.240
	*p*		0.570	0.950	0.787
Disease classification	Mild	79	39.90 ± 9.03	40.71 ± 6.74	80.61 ± 14.25
	Severe	39	38.05 ± 8.35	38.26 ± 7.72	76.31 ± 13.99
	*F*		1.146	3.136	2.406
	*p*		0.287	0.079	0.124
Self-conceived possibility of being-infected before see the doctor	Very high	17	41.65 ± 7.88	42.35 ± 5.11	84.00 ± 11.66
	Little high	18	41.56 ± 8.25	39.33 ± 6.62	80.89 ± 12.62
	General	18	41.33 ± 7.00	39.78 ± 6.50	81.11 ± 11.95
	Lower	37	40.54 ± 6.47	41.95 ± 4.77	82.49 ± 8.50
	Unclear	28	33.43 ± 11.11	36.14 ± 9.83	69.57 ± 19.58
	*F*		4.627	3.504	4.887
	*p*		0.002	0.010	0.001
Attention to the publicity of COVID-19	Very concerned	55	39.27 ± 8.75	39.42 ± 7.07	78.69 ± 13.58
	General attention	58	39.93 ± 8.16	40.97 ± 6.28	80.90 ± 12.94
	Less concerned	5	32.00 ± 14.97	32.80 ± 13.08	64.80 ± 27.62
	*F*		1.888	3.373	3.107
	*p*		0.156	0.038	0.049
Study time per day (h)	<1	75	39.89 ± 8.75	39.68 ± 7.37	79.57 ± 14.43
	1∼3	37	38.49 ± 8.81	40.32 ± 7.27	78.81 ± 14.48
	>3	6	36.67 ± 10.56	40.00 ± 2.53	76.67 ± 12.24
	*F*		0.590	0.100	0.132
	*p*		0.556	0.905	0.876

Note: ANOVA analysis was used to compare the mean score of disease-related knowledge among different groups of COVID-19 patients.

### Correlation Between Disease-Related Knowledge and General Status

Spearman correlation analysis showed that the self-care situation was positively correlated with disease prevention and control knowledge (*r* = 0.265; *P* = 0.004) and total score of disease-related knowledge (*r* = 0.206; *P* = 0.025); the degree of anxiety was negatively correlated with the disease diagnosis and treatment knowledge (*r* = −0.182; *P* = 0.049) (Table 3).

**Table 3.** Correlation among disease-related knowledge, general status, illness perception, and psychological status (*n* = 118)

Variables	Disease diagnosis and treatment knowledge		Disease prevention and control knowledge		Total disease-related knowledge	
	*r*	*P*	*r*	*P*	*r*	*P*
General condition						
Self-care situation	0.106	0.253	0.265**	0.004	0.206*	0.025
Appetite	0.088	0.342	0.021	0.825	0.060	0.518
Activity endurance	0.076	0.413	0.084	0.368	0.086	0.353
Sleep	0.158	0.087	0.023	0.805	0.113	0.224
Emotional state	0.098	0.293	0.045	0.628	0.096	0.299
Anxiety level	−0.182	0.049	−0.123	0.184	−0.177	0.056
Negative effects of disease	−0.112	0.227	−0.152	0.101	−0.157	0.089
Illness perception						
Course of disease	−0.007	0.942	−0.077	0.409	−0.046	0.624
Volatility	−0.228*	0.013	−0.228*	0.013	−0.251**	0.006
consequences	−0.210*	0.022	−0.145	0.116	−0.214*	0.020
Personal control	0.143	0.122	0.148	0.111	0.165	0.073
Treatment control	0.076	0.411	0.142	0.126	0.124	0.182
disease coherence	0.181*	0.049	0.193*	0.036	0.209*	0.023
Emotional statement	−0.208*	0.024	−0.211*	0.022	−0.242**	0.008
POMS						
Tension	−0.142	0.124	−0.188*	0.041	−0.187*	0.042
Anger	−0.140	0.13	−0.147	0.112	−0.169	0.068
Fatigue	−0.140	0.13	−0.214*	0.020	−0.197*	0.033
Depression	−0.192*	0.037	−0.251**	0.006	−0.248**	0.007
Vigor	−0.031	0.738	−0.085	0.359	−0.053	0.570
Confusion	−0.135	0.146	−0.172	0.063	−0.170	0.065
Self-esteem	0.161	0.083	0.144	0.119	0.184*	0.046
TMD	−0.122	0.188	−0.131	0.158	−0.146	0.114

Note: Spearman correlation was used to analyze the correlation among the study variables.

* *P* < 0.05 (2-tailed).

** *P* < 0.01 (2-tailed).

### Correlation Between Disease-Related Knowledge and Illness Perception

Spearman correlation analysis showed that the volatility, emotional statements were negatively correlated with the disease diagnosis and treatment knowledge, disease prevention and control knowledge, and total disease-related knowledge (*P* < 0.05). The disease coherence was positively correlated with the disease diagnosis and treatment knowledge, disease prevention and control knowledge, and total disease-related knowledge (*P* < 0.05). The consequences were negatively correlated with the disease diagnosis and treatment knowledge and the total score of disease-related knowledge (*P* < 0.05) (Table 3).

### Correlation Between Disease-Related Knowledge and Psychological Status

Spearman correlation analysis has shown that tension and fatigue were negatively correlated with the disease prevention and control knowledge and total score of disease-related knowledge (*P* < 0.05). Depression was negatively correlated with the disease diagnosis and treatment knowledge, disease prevention and control knowledge, and total score of disease-related knowledge (*P* < 0.05). Self-esteem was positively correlated with total score of disease-related knowledge (*P* < 0.05) (Table 3).

## Discussion

Our study confirmed that the disease diagnosis and treatment knowledge, disease prevention and control knowledge, and total disease-related knowledge of COVID-19 patients were at a upper-middle level, but there were still some patients who did not know enough about the COVID-19, and the score of disease diagnosis and treatment knowledge was lower than the disease prevention and control knowledge. This was consistent with the current situation. Before the outbreak of novel coronavirus pneumonia, the general public had no idea about the disease. After the epidemic broke out in January 2020, to control the situation as soon as possible, the Chinese government focused on the promotion of COVID-19 prevention and control knowledge online and offline nationwide,^26^ including the use of TV media for all-day broadcast. Meanwhile, the communities and government staff carried out the publicity education from door to door,^27^ which had increased the coverage and popularity of disease-related knowledge.

At the same time, with the exponential growth of confirmed cases in the early stages of the outbreak, people were generally willing to actively seek disease-related knowledge to protect themselves. Under the influence of multiple factors, the overall level of disease-related knowledge of COVID-19 patients was better. However, because these propaganda contents were mainly disease prevention and control, and the diagnosis and treatment plan had been continuously explored and improved, the level of disease diagnosis and treatment knowledge was lower than the disease prevention and control knowledge in this study. As there were some patients who still had a low level of disease-related knowledge, it was recommended to strengthen the health education for COVID-19 patients based on the existing situation, and increase the content of diagnosis and treatment of COVID-19 and self-care methods in the follow-up nursing process.

It was found that patients with higher family incomes and education levels, and those from 31 to 45 y of age had higher levels of disease-related knowledge than others, which were similar with the previous findings.^15,28^ This might be because young and middle-aged people and those with higher education and monthly income are more concerned about their health, have more access to disease-related knowledge, and have better understanding and memory of disease-related knowledge.

In addition, patients who have a high self-conceived possibility of being-infected before they see the doctor also have shown better disease-related knowledge. This might be because such patients have a clearer understanding of the disease infectiousness and pathogenicity based on their own conditions, and will more actively seek disease-related knowledge. Studies have also shown that people who pay close attention to the publicity of COVID-19 have higher levels of disease-related knowledge. The above results suggested that, when conducting health education in the future, medical staff should pay more attention to the elderly, low-education, and low-income patients, timely collect their feedback of training and education content, and effectively improve the patient’s disease-related knowledge level.

It was also shown that the higher the level of disease-related knowledge, the better the ability of self-care, and the lower the level of anxiety, which was consistent with previous results.^14,29^ This illustrated the importance of disease-related knowledge for COVID-19 patients during epidemic prevention and control. Anxiety is a manifestation of psychological stress. When the degree of uncontrollable and unpredictable incidents perceived by an individual is higher, the individual’s fear and anxiety will increase, causing irrational anxiety.^11^ As a public health emergency, COVID-19 is a major source of stress for patients.^5^ After patients acquire disease-related knowledge, they will consider the COVID-19 is preventable and controllable, which will help them to reduce anxiety and maintain rational behavior. This will also improve their treatment compliance and self-care ability, and increase their awareness of health, so that they can master the 7-step hand-washing method, wearing a mask, and daily disinfection methods to protect themselves and others from being infected. This result indicates that medical staff should promptly inform patients of the new progress of the current diagnosis and treatment, reduce negative emotion, and promote the cooperation and support of patients in treatment, nursing care, and self-isolation.

This study indicated that the more disease-related knowledge a patient had, the lower the disease-related negative cognition (volatility, consequences, and emotional statements), and the better the disease-related positive cognition (disease coherence). The positive cognition skills can be incorporated into cognitive behavior therapy, which was found to be effective to treat psychiatric conditions during the COVID-19 pandemic.^30^ Volatility and consequences refer to the repetitiveness and severity of the patient’s own disease and the extent to which the disease affects the patient’s physical and social functions; the emotional statement reflects the psychological pressure of the disease on the patient; the sense of disease agreement reflects the patients’ understanding of their disease.^18,31^

In previous studies, disease awareness was able to predict disease-related coping behaviors, such as seeking medical help and treatment compliance.^19,31^ Therefore, improving COVID-19 patients’ awareness of the disease can enhance their symptom recognition ability, enhance self-protection ability, and enable them to better cooperate with the hospital’s treatment and isolation protocols. While improving disease-related knowledge, it is recommended to carry out interventions based on the theory of knowledge-attitude-practice to improve patients’ and the general population’s awareness of the disease, thereby promoting healthy behaviors to achieve the purpose of controlling the source of infection, cutting off the route of transmission, and protecting vulnerable populations.

This study indicates that, as patients gain more disease-related knowledge, the fewer negative emotions they have, including tension, fatigue, and depression. Studies have shown that survivors of the SARS epidemic in 2003 suffered a series of emotional stresses such as anxiety, anger, nervousness, and depression.^32^ Under the stimulation of this emotional response and stressors, people will also form corresponding self-defensive mechanisms,^33,34^ such as denial mechanisms (denying illness, not cooperating with treatment), transfer mechanisms (not understanding isolation measures, anger at medical care), which can alleviate psychological pain to a certain extent, but they can prevent rational thinking and problem solving.^35^ As a public health emergency of the same type as SARS, the psychological state and defense mechanisms of COVID-19 patients are similar. Therefore, to reduce the impact of poor psychological defense mechanisms on patients, it is possible to enhance patients’ awareness of diseases by strengthening education on disease-related knowledge.

### Strengths and Limitations of the Study

Our research had some limitations. First, like all cross-sectional studies, this study could not explain the relationship between disease-related knowledge and the long-term effects of mental state and disease perception. It would be better to conduct a prospective study on these participants later. However, the ethical requirements of anonymity and confidentiality did not allow us to collect personal information of research participants, so this prospective study could not be conducted. Second, all demographic and main assessment indicators of the psychological states were reported by the patients themselves, which might lead to reporting bias. In addition, this study was conducted only in Hunan, China, and might not be representative of all COVID-19 patients. In future studies, it was necessary to expand the sample size and research sites to reduce these deviations. Notwithstanding the above limitations, our results provided valuable information for the relationship between disease-related knowledge, psychological status, and illness perception of COVID-19 patients. Most importantly, our findings could directly guide psychological cognition interventions, which could minimize the negative psychological impact and improve the knowledge of disease prevention and treatment and treatment of patients and ordinary people to maintain physical and mental health during the COVID-19 pandemic.

## Conclusions

COVID-19 is a public health emergency that poses a huge threat to people’s lives, health, and safety. With the joint efforts of the Chinese government and individuals, the current disease-related knowledge of COVID-19 patients was at a moderately high level. Universal health education still needs to be further strengthened, especially paying attention to the elderly and low-income groups, and the content should also increase the knowledge of diseases diagnosis and treatment and self-care. Based on the strong correlation between disease-related knowledge and the patient’s general condition, illness perception, and psychological status, future research can carry out interventions based on the theory of knowledge-attitude-practice to improve patients and the general population’s awareness of the disease. At the same time, the role of cognitive behavioral intervention and psychotherapy in reducing the psychological stress response can be explored to continuously promote the comprehensive physical and psychological recovery of the patients with COVID-19.

## Data Availability

The data that support the findings of this study are available from the corresponding author upon reasonable request.

## Supplementary material

For supplementary material accompanying this paper visit http://dx.doi.org/10.1017/dmp.2021.33. click here to view supplementary material

## References

[1] Hui DS , Azhar EI , Madani TA , et al. The continuing 2019-nCoV epidemic threat of novel coronaviruses to global health - the latest 2019 novel coronavirus outbreak in Wuhan, China. Int J Infect Dis. 2020;91:264–266. doi: 10.1016/j.ijid.2020.01.009 31953166 PMC7128332

[2] The National Health Commission of China. Updates on the novel coronavirus outbreak up to March 10, 2020. COVID-19 outbreak reports page. National Health Commission of the People’s Republic of China website. http://www.nhc.gov.cn/xcs/yqtb/202003/b4abcf83e53d4284b2981c75917385eb.shtml. Accessed March 11, 2020.

[3] Rothe C , Schunk M , Sothmann P , et al. Transmission of 2019-nCoV infection from an asymptomatic contact in Germany. N Engl J Med. 2020;382(10):970–971. doi: 10.1056/NEJMc2001468 32003551 PMC7120970

[4] World Health Organization (WHO). Coronavirus disease 2019 (COVID-19) situation report-52. World Health Organization website. https://www.who.int/docs/default-source/coronaviruse/situation-reports/20200312-sitrep-52-covid-19.pdf. Accessed March 12, 2020.

[5] Lu CW , Liu XF , Jia ZF. 2019-nCoV transmission through the ocular surface must not be ignored. Lancet. 2020;395(10224):e39. doi: 10.1016/s0140-6736(20)30313-5 32035510 PMC7133551

[6] Huang C , Wang Y , Li X , et al. Clinical features of patients infected with 2019 novel coronavirus in Wuhan, China. Lancet. 2020;395(10223):497–506. doi: 10.1016/s0140-6736(20)30183-5 31986264 PMC7159299

[7] Tan W , Hao F , McIntyre RS , et al. Is returning to work during the COVID-19 pandemic stressful? A study on immediate mental health status and psychoneuroimmunity prevention measures of Chinese workforce. Brain Behav Immun. 2020;87:84–92. doi: 10.1016/j.bbi.2020.04.055 32335200 PMC7179503

[8] Wu Z , McGoogan JM. Characteristics of and important lessons from the coronavirus disease 2019 (COVID-19) outbreak in China: summary of a report of 72314 cases from the Chinese Center for Disease Control and Prevention. JAMA. 2020;323(13):1239–1242. doi: 10.1001/jama.2020.2648 32091533

[9] Wang C , Pan R , Wan X , et al. A longitudinal study on the mental health of general population during the COVID-19 epidemic in China. Brain Behav Immun. 2020;87:40–48. doi: 10.1016/j.bbi.2020.04.028 32298802 PMC7153528

[10] Hao F , Tan W , Jiang L , et al. Do psychiatric patients experience more psychiatric symptoms during COVID-19 pandemic and lockdown? A case-control study with service and research implications for immunopsychiatry. Brain Behav Immun. 2020;87:100–106. doi: 10.1016/j.bbi.2020.04.069 32353518 PMC7184991

[11] Wang C , Pan R , Wan X , et al. Immediate psychological responses and associated factors during the initial stage of the 2019 coronavirus disease (COVID-19) epidemic among the general population in China. Int J Environ Res Public Health. 2020;17(5):1729. doi: 10.3390/ijerph17051729 PMC7084952 32155789

[12] Wu KK , Chan SK , Ma TM. Posttraumatic stress, anxiety, and depression in survivors of severe acute respiratory syndrome (SARS). J Trauma Stress. 2005;18(1):39–42. doi: 10.1002/jts.20004 16281194 PMC7166878

[13] Mak IW , Chu CM , Pan PC , et al. Long-term psychiatric morbidities among SARS survivors. Gen Hosp Psychiatry. 2009;31(4):318–326. doi: 10.1016/j.genhosppsych.2009.03.001 19555791 PMC7112501

[14] Molenaar S , Sprangers MA , Rutgers EJ , et al. Decision support for patients with early-stage breast cancer: effects of an interactive breast cancer CDROM on treatment decision, satisfaction, and quality of life. J Clin Oncol. 2001;19(6):1676–1687. doi: 10.1200/jco.2001.19.6.1676 11250997

[15] Yoo YS , Cho OH , Cha KS. Disease-related knowledge and information needs among inflammatory bowel disease patients in Korea. Gastroenterol Nurs. 2015;38(6):455–463. doi: 10.1097/sga.0000000000000063 25159269 PMC4666008

[16] Tran BX , Dang AK , Thai PK , et al. Coverage of health information by different sources in communities: implication for COVID-19 epidemic response. Int J Environ Res Public Health. 2020;17(10):3577. doi: 10.3390/ijerph17103577 PMC7277747 32443712

[17] Casellas F , Fontanet G , Borruel N , et al. The opinion of patients with inflammatory bowel disease on healthcare received. Rev Esp Enferm Dig. 2004;96(3):174–184. doi: 10.4321/s1130-01082004000300003 15053732

[18] Bassi M , Grobberio M , Negri L , et al. The contribution of illness beliefs, coping strategies, and social support to perceived physical health and fatigue in multiple sclerosis. J Clin Psychol Med Settings. 2019. doi: 10.1007/s10880-019-09692-6. [Epub ahead of print]. 31872372

[19] Velez-Velez E , Bosch RJ. Illness perception, coping and adherence to treatment among patients with chronic kidney disease. J Adv Nurs. 2016;72(4):849–863. doi: 10.1111/jan.12873 26689295

[20] Weinman J , Petrie KJ , Moss-Morris R , et al. The illness perception questionnaire: a new method for assessing the cognitive representation of illness. Psychol Health. 1996;11(3):431–445. doi: 10.1080/08870449608400270

[21] Moss-Morris R , Weinman J , Petrie K , et al. The Revised Illness Perception Questionnaire (IPQ-R). Psychol Health. 2002;17(1):1–16. doi: 10.1080/08870440290001494

[22] Xiong NN , Wei J , Hong X , et al. Cross-culture adaptation, validity and reliability of the Chinese version of the Illness Perception Questionnaire-Revised (IPQ-R) among outpatients. Chin J Ment Health. 2018;32(09):713–719.

[23] Perciavalle V , Blandini M , Fecarotta P , et al. The role of deep breathing on stress. Neurol Sci. 2017;38(3):451–458. doi: 10.1007/s10072-016-2790-8 27995346

[24] Zhu PL. Brief introduction of POMS scale and its model for China. J Tianjin Inst Physical Educ. 1995;(01):35–37.

[25] Loh KP , Kleckner IR , Lin PJ , et al. Effects of a home-based exercise program on anxiety and mood disturbances in older adults with cancer receiving chemotherapy. J Am Geriatr Soc. 2019;67(5):1005–1011. doi: 10.1111/jgs.15951 31034591 PMC6544022

[26] Ye Q , Wang B , Mao J , et al. Epidemiological analysis of COVID-19 and practical experience from China. J Med Virol. 2020;92(7):755–769. doi: 10.1002/jmv.25813. 32237160 PMC7228220

[27] Liu W , Yue XG , Tchounwou PB. Response to the COVID-19 epidemic: the Chinese experience and implications for other countries. Int J Environ Res Public Health. 2020;17(7):2304. doi: 10.3390/ijerph17072304 PMC7177503 32235413

[28] Tran BX , Vu GT , Latkin CA , et al. Characterize health and economic vulnerabilities of workers to control the emergence of COVID-19 in an industrial zone in Vietnam. Saf Sci. 2020;129:104811. doi: 10.1016/j.ssci.2020.104811 32398902 PMC7214303

[29] Song P , Karako T. COVID-19: real-time dissemination of scientific information to fight a public health emergency of international concern. Biosci Trends. 2020;14(1):1–2. doi: 10.5582/bst.2020.01056 32092748

[30] Ho CS , Chee CY , Ho RC. Mental health strategies to combat the psychological impact of COVID-19 beyond paranoia and panic. Ann Acad Med Singapore. 2020;49(3):155–160. 32200399

[31] Mosleh SM , Almalik MM. Illness perception and adherence to healthy behaviour in Jordanian coronary heart disease patients. Eur J Cardiovasc Nurs. 2016;15(4):223–230. doi: 10.1177/1474515114563885 25505161

[32] Lee AM , Wong JG , McAlonan GM , et al. Stress and psychological distress among SARS survivors 1 year after the outbreak. Can J Psychiatry. 2007;52(4):233–240. doi: 10.1177/070674370705200405 17500304

[33] Cramer P. Defense mechanisms in psychology today. Further processes for adaptation. Am Psychol. 2000;55(6):637–646. doi: 10.1037//0003-066x.55.6.637 10892206

[34] Babl A , Grosse Holtforth M , Perry JC , et al. Comparison and change of defense mechanisms over the course of psychotherapy in patients with depression or anxiety disorder: evidence from a randomized controlled trial. J Affect Disord. 2019;252:212–220. doi: 10.1016/j.jad.2019.04.021 30986736

[35] Liang BY. Common psychological stress response and psychological intervention during the SARS epidemic. Stud Psychol Behav. 2003;(03):223–230.

