# Supplementary material for: Effects of Disease-Related Knowledge on Illness Perception and Psychological Status of Patients With COVID-19 in Hunan, China
Source: Disaster Med Public Health Prep. 2021 Feb 16:1–8. doi: 10.1017/dmp.2021.33 (PMC8010286; doi:10.1017/dmp.2021.33)
Supplement: Supplementary file 1 [file S1935789321000331sup001.docx]

Disease-Related Knowledge Questionnaire of COVID-19

The following is the relevant knowledge of COVID-19, according to the actual situation you know, tick the option you think is correct (correct = 4 points, wrong = 0 points)

1. (Etiological characteristics) Which of the following cannot inactivate the 2019 novel coronavirus (2019-nCoV)? (D)

A. Ultraviolet radiation

B. Maintain for 30 minutes at 56 ℃

C. 75% alcohol

D. Chlorhexidine

E. Chlorine disinfectant

2. (Epidemiological characteristics) Except for any of the following animals, it is possible to carry the 2019-nCoV? (C)

A. Civet

B. Bat

C. Mosquito

D. Bamboo rat

E. Badger

3. (Route of transmission) Which of the following is not included in the known routes of transmission of the 2019-nCoV? (C)

A. Droplet transmission

B. Contact transmission

C. Insect-borne spread

D. Aerosol propagation

E. Alimentary tract transmission

4. (Incubation period / quarantine period) How many days does a person need medical observation after leaving the quarantine? (D)

A. 3 days

B. 1 week

C. 8 days

D. 14 days

E. 30 days

5. (Susceptible and high-risk population) Which type of population is not at high-risk population of COVID-19? (C)

A. Pregnant women

B. Children under 5 years old

C. Young adults

D. Old people over 60 years old

E. Patients with chronic diseases

6. (Clinical manifestations) Which is incorrect about the clinical manifestations of COVID-19? (E)

A. Clinical symptoms mainly include fever, fatigue, and dry cough

B. A few patients have symptoms such as nasal congestion, runny nose, sore throat, and diarrhea

C. Most severe patients develop dyspnea and / or hypoxemia after 1 week

D. Critical patients will have respiratory failure, shock or organ dysfunction

E. There must be clinical manifestations of pneumonia

7. (Diagnosis and medical treatment) If you are infected with the 2019-nCoV, the correct approach is? (A)

A. Take the initiative to wear a mask and seek medical treatment nearby

B. Continue to work or go to school with illness

C. Escape as soon as possible to other virus-free areas

D. Never go to the hospital

E. Endure at home

8. (Treatment)Which of the following treatments for COVID-19 is wrong? (D)

A. Bed rest

B. Oxygen inhalation

C. Antiviral therapy

D. Routine antibacterial treatment

E. Psychological counseling

9. (Psychological adjustment) Anxiety and fear are normal reactions after your diagnosis, which of the following measures is scientific and can relieve anxiety? (B)

A. Wash your hands every 1 or 2 hours

B. Make a regular life plan and find things that will make you happy and relaxed

C. Pay more attention to and browse the epidemic

D. Reunion with family

E. Ask the doctor to do nucleic acid test every 2 days

10. (Nursing) For patients with mild COVID-19 during isolation observation, which of the following is inappropriate? (D)

A. Do not use central air conditioning during hospitalization

B. Ensuring adequate rest

C. Do not leave the isolation room at will

D. Keep the door open continuously for ventilation

E. Maintain a nutritious diet and enough calories

11. (Prognosis) What is incorrect about the prognosis of COVID-19? (A)

A. Most patients have a poor prognosis

B. A few patients are in critical condition

C. The elderly has a poor prognosis

D. The prognosis of people with chronic underlying diseases will be worse

E. Children have relatively mild symptoms

12. (Discharge criteria) When can a diagnosed patient be released from isolation or discharged after treatment? (E)

A. Body temperature recovered for more than 3 days

B. Respiratory symptoms improved significantly

C. Two consecutive negative respiratory tract nucleic acid tests (sampling interval at least 1 day)

D. Chest CT showed normal lung imaging

E. With all the conditions of A, B, and C

13. (Epidemiology / Law-related) Which of the following practices when seeking medical treatment violates the "Infectious Disease Control Act"? (E)

A. People should proactively report to the community their travel history of Wuhan and surrounding areas or other cases

B. People should take the initiative to report to the doctor that there is a similar situation in the family

C. People should actively report to doctors that they have been in contact with suspicious cases

D. People should actively report to doctors that they have been in contact with confirmed cases

E. People should conceal their recent activity routes and suspected symptoms of new coronary pneumonia

14. (Home isolation) When someone is determined by the hospital as a suspected case of COVID-19 and requires home self-isolation, which of the following practices is incorrect? (C)

A. Drink more water during isolation

B. Try to keep more than 1 meter away from family

C. Participate in family dinners and share tableware

D. Clean all high-frequency contact surfaces every day

E. Put rubbish contaminated with mouth and nose secretions in covered trash can

15. (Home isolation) For those diagnosed with close contact with COVID-19, which of the following suggestions is incorrect? (E)

A. They should receive medical observation for 14 days after the last contact with the confirmed case

B. During the observation period, they should not go out, live in a single room, and keep the distance of more than 1 meter between family members

C. During the observation period, the body temperature should be measured every day. When fever, cough, sore throat, dyspnea, and other symptoms occur, go to the fever clinic as soon as possible

D. Garbage bins with lids and special garbage bags should be prepared in the room. Personal garbage should be sealed and discarded after entering the garbage bags

E. No need to wear a mask when they are in the same room with their family

16. (Prevention and control) Which of the following statements about the droplet spread is incorrect? (D)

A. Close contact with the person carrying the virus, the virus can be directly transmitted to the susceptible

B. Public places such as elevator cabs or crowded meeting rooms are high-risk locations

C. Talking, coughing, and sneezing can cause droplet spread

D. Medical masks cannot stop the droplet spread

E. Spitting can cause droplet spread

17. (Prevention and control) Which of the following practices is incorrect when coughing or sneezing? (C)

A. Cover your nose and mouth with paper towels

B. Cover your mouth and nose with your elbows

C. Cover the mouth and nose with both hands

D. Put the used tissues in the covered trash can

E. Clean your hands after coughing and sneezing

18. (Prevention and control) Which of the following masks is generally used to block the spread of respiratory secretions? (B)

A. Paper mask

B. Disposable medical masks

C. Cotton mask

D. Activated carbon mask

E. Sponge mask

19. (Prevention and control) Which of the following statements about wearing masks to prevent the spread of respiratory diseases is incorrect? (D)

A. It is recommended to replace it every 2-4 hours

B. Replace it as soon as it becomes contaminated

C. When wearing, avoid touching the inside of the mask with your hands

D. The thicker the mask, the better the anti-virus effect

E. Used masks cannot be disinfected with alcohol and then reused

20. (Prevention and control) Which of the following measures to prevent COVID-19 is inappropriate? (D)

A. Exercise properly

B. Early to bed and get up early

C. All rooms must be opened at least twice a day for ventilation, and more than half an hour each time

D. Put white vinegar on the radiator to disinfect the indoor air

E. Adhere to a safe diet and eat meat and eggs cooked

21. (Prevention and control) Which of the following is correct about the use and handling of masks? (A)

A. Always wash your hands before wearing a mask, and avoid touching the inside of the mask

B. You can touch the mask during use

C. Grasping the outside of the mask when taking off the mask

D. No need to wear masks in a closed place with low population density

E. Take off the mask and put it directly in your pocket or bag

22. (Prevention and control) Which of the following behaviors is incorrect when going home? (E)

A. Take off your outerwear and replace it with leisurewear

B. Take off the mask and throw it in the trash

C. The mask removed should not be placed randomly

D. Wash hands and disinfect after processing masks and clothing

E. Close the window to prevent outside air from entering the room

23. (Prevention and control) What are the following ways to effectively prevent the COVID-19? (B)

A. Gargle with light saline

B. Wear a mask

C. Sauna and steam

D. Chewing betel nut

E. Drinking alcohol

24. (Prevention and control) Which of the following can enhance immunity and prevent COVID-19? (E)

A. Do not go to crowded places

B. Wear a mask

C. Wash hands frequently

D. Maintain air circulation indoors

E. Reasonable work and rest, adequate sleep, pay attention to nutrition, and keep exercising

25. (Prevention and control) Which of the following circumstances does not require washing hands? (E)

A. After going to the toilet

B. After coughing and sneezing

C. Before, during and after food preparation

D. After contact with animals

E. Before handling feces
